# Identification of Novel Recombinant Human Adenovirus Genotype B117 from Pediatric Cases, China

**DOI:** 10.3201/eid3206.250940

**Published:** 2026-06

**Authors:** Jinjin Wang, Ling Jing, Yali Duan, Xiaolei Guan, Yue Cui, Junhong Ai, Ran Wang, Xiangpeng Chen, Yuhai Bi, Xiaomei Liu, Baoping Xu, Yun Zhu, Zhengde Xie

**Affiliations:** Beijing Children’s Hospital, Capital Medical University, Beijing, China (J. Wang, Y. Duan, X. Guan, Y. Cui, J. Ai, R. Wang, X. Chen, X. Liu, B. Xu,Y. Zhu, Z. Xie); Chinese Academy of Medical Sciences & Peking Union Medical College, Beijing (L. Jing); Chinese Academy of Sciences, Beijing (Y. Bi).

**Keywords:** human adenovirus, recombination, genotype, community-acquired pneumonia, children, pediatrics, viruses, viral infections, China

## Abstract

During molecular surveillance of human adenoviruses (HAdVs) in children hospitalized with acute lower respiratory tract infections in Beijing, China, during 2014–2024, the most prevalent genotypes were HAdV-B114 (53.85%) and HAdV-B7 (27.18%). A novel recombinant genotype, HAdV-B117, was identified in 2 children <5 years of age with severe community-acquired pneumonia and serious complications. Genomic analysis revealed that HAdV-B117 arose from HAdV-B114 (P7H3F3) with the fiber gene from HAdV-B7. We observed amino acid substitutions and deletions in the pivotal regions of 3 major capsid proteins, and some were predicted to alter the protein structure. In vitro, the replication kinetics of HAdV-B117 were similar to those of HAdV-B3 and HAdV-B7. Clinical manifestations resembled severe pneumonia caused by HAdV-B3 or HAdV-B7. Both children recovered after treatment. The emergence of HAdV-B117 highlights the need for continuous genomic surveillance of HAdVs to detect novel recombinants with potential public health effects.

Human adenoviruses (HAdVs) are nonenveloped, double-stranded DNA viruses of the genus *Mastadenovirus*, Adenoviridae family (*1*). To date, 116 genotypes have been recognized within 7 species (A–G) (http://hadvwg.gmu.edu). Currently, the nomenclature protocol primarily follows the system proposed by the international HAdV Working Group (*2*), which defines genotypes on the basis of the nucleotide sequences of the penton base, hexon, and fiber genes. HAdV-B (HAdV-3, -7, -55), HAdV-C (HAdV-1, -2, -5, -6), and HAdV-E (HAdV-4) are considered major causative agents of acute respiratory tract infections in children (*3*). Within species B, HAdV-B3 and HAdV-B7 are the most frequently reported etiologic agents worldwide and are associated with severe pneumonia and occasional fatalities in pediatrics (*4*,*5*). Homologous recombination, particularly within the 3 major capsid protein genes, largely contributes to HAdV-B diversity, yielding recombinant genotypes with high virulence and broad tissue tropism (*6*), some of which have caused severe outbreaks (e.g., HAdV-B55, HAdV-B66) (*7*–*10*).

Densely populated and highly mobile metropolises are hotspots for HAdV transmission. However, in China, the disease effects of HAdVs, including HAdV-B, remain unclear because of the absence of a specialized surveillance system (*11*). We conducted a molecular epidemiologic investigation of HAdVs in Beijing, China, during 2014–2024. By applying whole-genome sequencing (WGS) to HAdV isolates from children hospitalized with acute lower respiratory tract infections (LRTIs) and analyzing genomic data, a novel intertypic recombinant HAdV-B genotype was identified from 2 children <5 years of age with severe community-acquired pneumonia (CAP). According to the evaluation of the international HAdV Working Group, the virus was designated HAdV-117 (P7H3F7). The identification of the novel recombinant HAdV-B117 underscores the need for ongoing genomic surveillance to monitor emerging HAdV threats.

## Materials and Methods

### Patients and Clinical Data Collection

Children <18 years of age hospitalized with acute RLTI at Beijing Children’s Hospital, Capital Medical University, Beijing, China, during 2014–2024 were enrolled. Children with a disease course >7 days or whose parents or guardians did not consent were excluded. We collected clinical data from the clinical electronic medical record system.

### Specimen Collection and HAdV Detection

Respiratory specimens, including sputum, throat and nasopharyngeal swab specimens, bronchoalveolar lavage fluid, and nasopharyngeal aspirates, were collected within 24 hours of admission and transported at 4°C to our laboratory. We tested all samples by using the Multiplex Respiratory Viral Panel (Diasorin, https://us.diasorin.com) for the presence of viral nucleic acids, including HAdV and other common respiratory viruses.

### Virus Isolation and WGS

We inoculated HAdV-positive samples into Hep-2 or A549 cell lines for virus isolation and purified the novel genotype HAdV through 3 rounds of plaque purification in A549 cells. We sent the purified nucleic acids of the HAdV strains to Annaroad Gene Technology Co., Ltd (Beijing, China, https://www.annoroad.com) for WGS by using the Illumina sequencing platform (Illumina, https://www.illumina.com). We used reference-based assembly to construct consensus sequences. We determined the reference genomes by the traditional HAdV typing method as previously described (*12*). We used the HAdV-B3 prototype strain (the GB strain, GenBank accession no. AY599834) as the reference genome of the novel genotype HAdV. If >2 distinct genomic sequences >34 kb were obtained from the same strain, we considered the possibility of co-infection. We annotated complete genome sequences by using Prokka version 1.11 (https://github.com/tseemann/prokka) and submitted them to GenBank.

### HAdV Typing

We extracted the penton base, hexon, and fiber gene sequences from the HAdV whole-genome sequences obtained and aligned them with sequences in GenBank by using BLAST (https://blast.ncbi.nlm.nih.gov) for preliminary typing. We retrieved the reference sequences for the 3 genes of HAdV from GenBank, excluding those with high consistency in region, time, and sequence. We performed multiple sequence alignment by using MAFFT (https://www.ebi.ac.uk/Tools/msa/mafft). We constructed phylogenetic trees by using MEGA version 7.0.26 (https://www.megasoftware.net/older_versions) with the neighbor-joining method and Kimura 2-parameter model, with a bootstrap test of 1,000 replicates to further determine the type.

### Phylogenetic Analysis

We selected whole-genome sequences of prototype or representative strains of all genotypes within the same species as the candidate novel genotype from GenBank. On the basis of their annotations, we extracted the penton base, hexon, fiber, and early gene (*E1–E4*) gene sequences for phylogenetic analysis. We constructed the phylogenetic trees by using the maximum-likelihood method with 1,000 bootstrap replicates in MEGA version 7.0.26 after multiple sequence alignments. We considered the model with the lowest Bayesian information criterion score optimal.

### Recombination Analysis

We conducted recombination analysis of the whole-genome sequences of the novel genotype by using RDP4 (https://www.h3abionet.org/categories/blog-post/tools-services/recombination-detection-program) with 7 methods (RDP, GENECONV, BootScan, MaxChi, Chimaera, SiScan, and 3Seq). We corrected the probability of each putative recombination event by using the Bonferroni procedure and set the significance threshold at p<0.05. We considered credible only recombination events supported by >3 methods. We validated the identified recombination events by using SimPlot version 3.5.1 (https://sray.med.som.jhmi.edu/SCRoftware/SimPlot) using window size 1,000, step size 200, gap stripping turned on, and Kimura 2-parameter distance correction.

### Genetic Variation Analysis

We used BioEdit version 7.2.05 (http://www.mbio.ncsu.edu/BioEdit) to perform genetic variation analysis. We constructed homology models of proteins by using Python version 3.10.7 (https://www.python.org) and Swiss-Model ExPASy (https://swissmodel.expasy.org).

### Ethics Considerations

This study was approved by the Institutional Ethics and Review Committees of the Beijing Children's Hospital, Capital Medical University (ethics approval nos. [2023]-E-171-Y, 2019-k-357), and conducted in accordance with Helsinki Declaration (revised in 2013). Written informed consent was obtained from all parents or legal guardians of children included.

## Results

### Detection Rate of HAdV

A total of 3,875 children with acute RLTI were enrolled in this study; 310 (8%) tested positive for HAdV. Viral isolation and genotyping were successfully achieved for 195 of the HAdV-positive specimens. The identified viruses included species B (HAdV-3, -7, -14, -21, -55, -66, and -114) and C (HAdV-1, -6, -89, and -108). The most common genotype was HAdV-114 (105 isolates, 53.85%), followed by HAdV-7 (53 isolates, 27.18%), HAdV-1 (12 isolates, 6.15%), and HAdV-108 (11 isolates, 5.64%). We identified a novel HAdV-B genotype (2 isolates, 1.03%).

### Clinical Manifestations of Patients with the Novel HAdV-B Genotype

The clinical characteristics of 2 cases infected with the novel HAdV-B genotype are provided (Appendix Table 1). Both patients were diagnosed with severe CAP. The first patient, a 4-year-old boy living in Beijing, China, with allergic rhinitis, was admitted to the general ward on December 19, 2019, and had high fever (40°C, reference range 36°C–37.2°C), cough, and sputum secretion. The leukocyte count was unremarkable, and C-reactive protein and erythrocyte sedimentation rate were mildly elevated. Chest radiograph and computed tomography (CT) both suggested pneumonia. Adenovirus nucleic acid was detected in throat swab specimens, plasma, and bronchoalveolar lavage fluid, and the adenovirus antigen test of respiratory secretions was positive. The patient’s complications included liver dysfunction and sinus bradycardia. Atelectasis was observed >1 month later. The patient received intravenous immunoglobulin, methylprednisolone, bronchoalveolar lavage, and antimicrobial drug therapy. The second patient was a 3-year-old girl from Hebei, China, with a patent foramen ovale. She was admitted to the respiratory ward on December 26, 2019, with a high fever (40.7°C), cough, sputum secretion, and slight tachypnea. The leukocyte count was unremarkable, and C-reactive protein, erythrocyte sedimentation rate, procalcitonin, and ferritin were increased. Chest radiograph and CT also suggested pneumonia. Adenovirus was detected in throat swab specimens, plasma, and nasopharyngeal swab specimens. Complications such as liver function damage, myocardial damage, and coagulopathy developed, and the patient received intravenous immunoglobulin, methylprednisolone, bronchoalveolar lavage, and antiviral therapy. Both patients fully recovered after treatments.

### Novel HAdV Genotype Identification

We isolated 2 HAdV strains (BJ-2024-563/2019 and BJ-2024-565/2019). BLAST alignment and phylogenetic analysis of penton base, hexon, and fiber genes revealed no matches with the 116 known HAdV genotypes. WGS of each strain yielded a single contiguous sequence of >34 kb, which we annotated and deposited in GenBank (accession nos. PV092676 [BJ-2024-563/2019] and PV092675 [BJ-2024-565/2019]). Both genomes are 35,299 bp with 50.83% guanine and cytosine content, indicating classification within species HAdV-B (49%–51% guanine and cytosine content).

### Phylogenetic Analysis of the Whole Genome and 3 Major Capsid Protein Genes

The whole-genome sequences of the 2 novel strains shared 99.7% nucleotide (nt) identity and 99.6% amino acid (aa) identity, with a genetic distance of 0.001. The penton base, hexon, and fiber gene sequences of the 2 strains exhibited 98.8%–99.9% nt identity and 97.5%–100% aa identity, with genetic distances ranging from 0.000–0.007. We retrieved reference sequences for the whole genome and penton base, hexon, and fiber genes of other genotypes of species HAdV-B from GenBank (Appendix Table 2).

**Figure 1:**
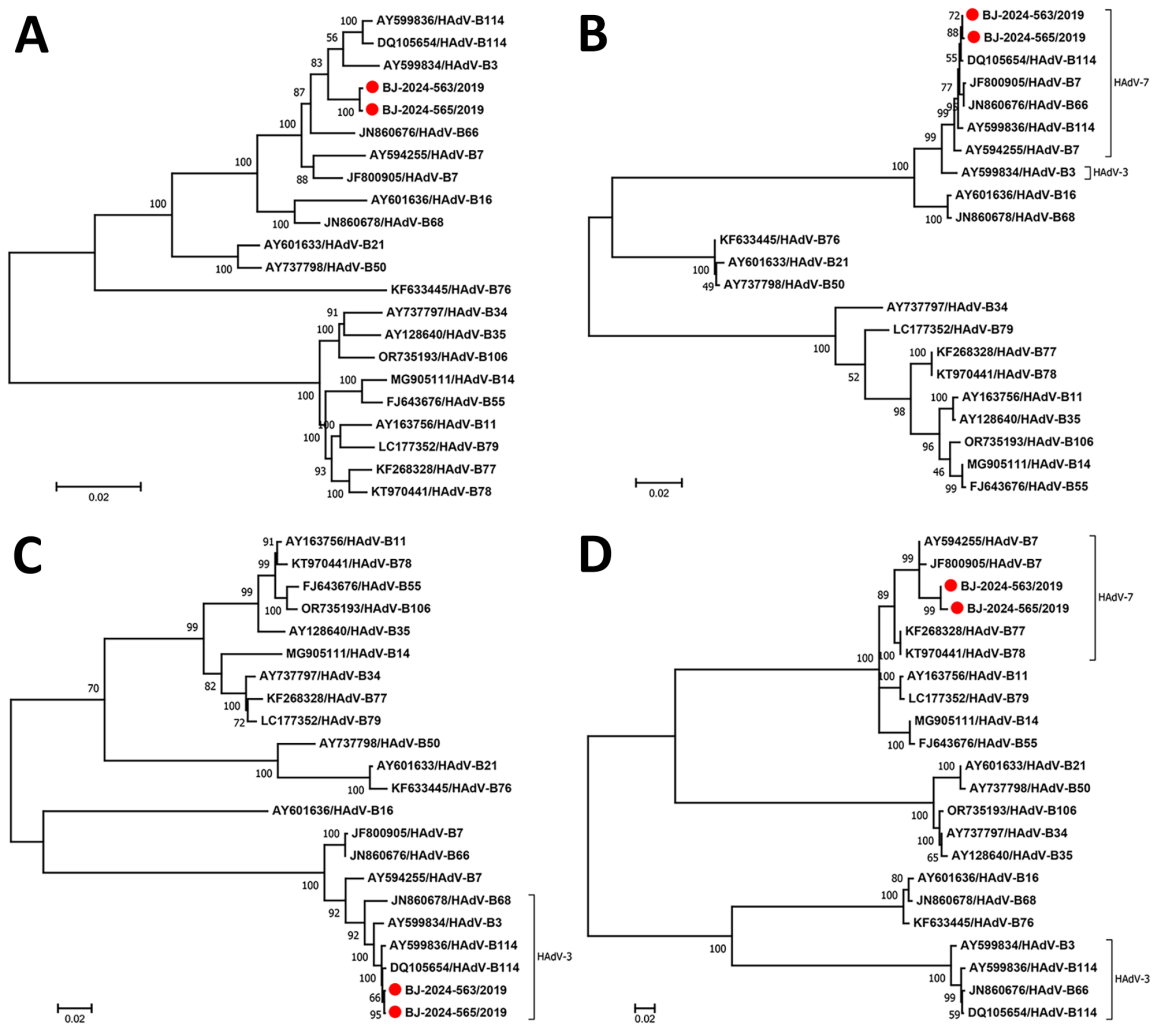
Phylogenetic analysis of the whole genome and 3 major capsid protein genes of 2 novel strains and other species HAdV-B genotypes from a study of novel recombinant human adenovirus genotype B117 from 2 pediatric cases, China. A) The phylogenetic tree of the whole genome, constructed by the general time reversible model. B) The phylogenetic tree of the penton base gene, constructed by the Kimura 2-parameter model. C, D) Phylogenetic trees of hexon (C) and fiber (D) genes, constructed by the Hasegawa-Kishino-Yano model. All trees were constructed by the maximum-likelihood method by using 1,000 replicates. Red dots indicate the 2 novel strains obtained in this study. Scale bars indicate nucleotide substitutions per site. HAdV, human adenovirus.

Phylogenetic analysis of the whole-genome sequences confirmed that the 2 novel strains belonged to the species HAdV-B and clustered together with a bootstrap value of 100%, forming a new evolutionary branch distinct from other known HAdV-B genotypes (Figure 1, panel A). Analysis of the penton base gene showed clustering with HAdV-B114 (88% bootstrap support) within the HAdV-7 evolutionary branch, including HAdV-B7 and HAdV-B66 (Figure 1, panel B). For the hexon gene, the strains grouped with HAdV-B114 (66% bootstrap support) within the HAdV-3 evolutionary branch, including HAdV-B3 and HAdV-B68 (Figure 1, panel C). The fiber gene phylogenetic analysis revealed clustering with HAdV-B7 (99% bootstrap support) within the HAdV-7 evolutionary branch, alongside HAdV-B77 and HAdV-B78 (Figure 1, panel D). The nt and aa identity analyses revealed that the whole genome, penton base, and hexon genes of the novel strains had high similarity with those of HAdV-B114, whereas the fiber gene aligned closely with HAdV-B7 (Table). Given that HAdV-B114 is a known recombinant genotype (P7H3F3) (*13*) and adhering to the current HAdV naming convention on the basis of the 3 major capsid protein genes (*2*), the 2 strains were proposed as a novel recombinant genotype, HAdV-B117, designated P7H3F7.

**Table:** Nucleotide and amino acid between BJ-2024-563/2019 HAdV-B isolate and counterparts from other HAdV-B genotypes from study of novel recombinant human adenovirus genotype B117 from 2 pediatric cases, China*

Genotype	Nucleotide/amino acid identity, %							
	Whole genome	Penton base	Hexon	Fiber	E1	E2	E3	E4
HAdV-3	96.8/95	98.5/99	97.4/97.3	64.3/57.3	96.9/94.2	98.2/96.4	94.7/92.1	98.5/97.5
HAdV-7	97.2–97.7/ 95.4–95.8	99.3–99.6/ 99.4–99.6	95.4–97.2/ 96.2–97.8	97.2–97.9/ 96.6–97.8	97.7–98.4/ 95.3–96	85.8–98.2/ 83.1–96.5	95.8–97.2/ 92.4–94.1	97.9–99.1/ 95.1–97.9
HAdV-11	82.9/73.1	79.5/83.5	78.5/85.3	92.5/91.3	80.6/57.4	55.9/49.2	76.6/63.6	95.5/90.1
HAdV-14	82.6/72.4	80.4/83.6	78/84.7	91.6/89.8	79.8/55.7	73.3/61.5	76.3/62.8	95.5/89.8
HAdV-16	93.6/91.2	93.7/94.4	79.9/86.5	59/52.8	96.8/93.4	96.6/93.1	95.3/92.6	72.3/65.8
HAdV-21	92.4/88.7	83.7/85.6	80.6/85.5	65.5/58.7	93.8/89.7	65/63.3	87.7/85.2	95.2/93.1
HAdV-34	82.2/72	79.9/83.5	77.4/84.2	66.4/60.2	77.8/54.5	79.9/66.2	69.7/58.2	93/88.3
HAdV-35	82.1/72.1	79.5/83.3	78/84.7	66.2/59.9	78/55.1	55.8/49.1	45.9/37.7	83.9/80.7
HAdV-50	92.4/88.7	83.4/85.4	81.5/86	65.4/58.4	96.1/92.3	83/75.8	96/93.2	98.2/96.3
HAdV-55	82.6/72.5	80.3/83.3	78.7/85	91.2/88.8	68.9/48.3	55.6/48.8	76.2/62.6	95.3/89.8
HAdV-66	96.1/94.4	99.6/99.8	95.5/96.3	65/58.5	95.4/94.1	85.8/83.3	88.5/86.3	95.6/94.8
HAdV-68	94.9/92.2	93.7/94.2	96/97.2	59/52.5	93.8/89.8	85.6/82.7	88.1/85.3	90.4/81.9
HAdV-76	85.2/76.1	83.8/85.8	79.8/85.1	58.7/53.3	83.7/65.8	79.4/70.6	75.8/65.4	79/60.8
HAdV-77	82.7/72.9	80/83.8	77.7/84.2	94.6/92.5	77.9/54.9	74.1/62.2	69.7/57.9	93.1/88.6
HAdV-78	83.1/73.3	80/83.8	78.3/84.9	94.6/92.5	78.2/55.5	74/62.3	76.7/63.4	93.2/89.1
HAdV-79	82.9/73	80/83.8	77.4/84.1	92.4/91.3	80.6/57.7	74/62.2	69.7/57.9	93.1/88
HAdV-106	82.3/72.3	80/83.5	78.4/85	66.3/59.9	78.2/55.2	55.8/49	69.7/58.2	84.2/80.9
HAdV-114	98–98.2/ 97.1–97.4	99.6–99.8/ 99.4–99.8	98.1–98.2/ 97.6–97.7	64.8–65/ 58.2–58.5	98.1–99.7/ 97.5–99.4	88.1–99.7/ 87–99.3	96.4–96.6/ 94.6–94.8	99.3–99.5/ 98.7–99

*There are high nucleotide and amino acid identities of the whole genome, penton base, hexon, fiber and E1–E4 sequences of the 2 novel strains obtained in this study, and according to nucleotide and amino acid identity analyses, the results were coincident. For this reason, BJ-2024-563/2019 was chosen as the representative to show the results. HAdV, human adenovirus.

### Phylogenetic Analysis of Early Genes

The early gene (*E1*–*E4*) sequences of the 2 strains exhibited 99.7%–99.8% nt identity and 99.4%–99.8% aa identity, with genetic distances of 0.000–0.001. Phylogenetic trees consistently showed clustering with HAdV-B114, supported by bootstrap values of 89%–100% (Appendix Figure 1). Identity analyses confirmed that the *E1*–*E4* genes were most similar to those of HAdV-B114 (Table). Specifically, the phylogenetic tree of *E1* genes revealed that the 2 sequences clustered together with HAdV-B114, -66, -7, and -3. HAdV-B16/21/50/68, HAdV-B14/34/55/79/106, and HAdV-B35/77/78 each formed a distinct cluster (Appendix Figure 1, panel A). Phylogenetic analysis of *E2* genes revealed that the 2 sequences clustered together with HAdV-B114. HAdV-B14/55/79/106 and HAdV-B11/34/35/77/78 clustered separately (Appendix Figure 1, panel B). The phylogenetic tree on the basis of *E3* genes revealed that the 2 sequences clustered together with HAdV-B114 and -3. The remaining genotypes grouped into 5 clusters: HAdV-B16/21/50, HAdV-B7/66/68, HAdV-B14/55, HAdV-B77/78, and HAdV-B11/34/35/79/106 (Appendix Figure 1, panel C). The phylogenetic analysis of *E4* genes revealed that the 2 sequences clustered with HAdV-B114, -7, and -66. HAdV-B21/50, HAdV-B14/55, HAdV-B11/34/35/77/78/79/106, and HAdV-B16/68 clustered together separately (Appendix Figure 1, panel D). Those findings indicate that the early gene regions of HAdV-B also harbored recombination hotspots.

### Recombination Analysis

**Figure 2:**
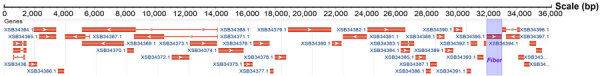
Genetic organization of novel HAdV-B117 isolate from a study of novel recombinant HAdV-B117 from 2 pediatric cases, China. HAdV, human adenovirus.

**Figure 3:**
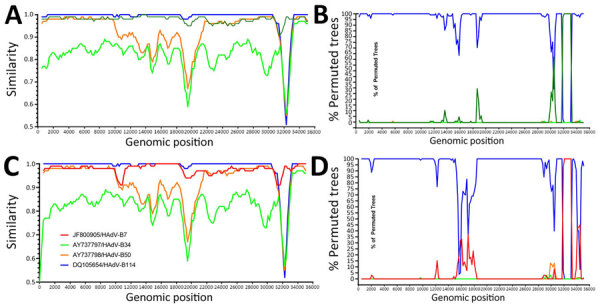
Recombination analysis between 2 novel strains and other species HAdV-B genotypes from a study of novel recombinant human adenovirus genotype B117 from 2 pediatric cases, China. A, B) SimPlot (A) and BootScan (B) analysis of the whole genome of the BJ-2024-563/2019 strain. C, D) SimPlot (C) and BootScan (D) analysis of the whole genome of the BJ-2024-565/2019 strain. HAdV, human adenovirus.

Recombination analysis by using RDP4 predicted 3 recombination events for the BJ-2024-563/2019 strain and 2 for the BJ-2024-565/2019 strain (Appendix Table 3). Simplot confirmed 2 events, establishing both strains as recombinants of HAdV-B7 and HAdV-B114. The genomic backbone corresponded to the Guangzhou02 strain (HAdV-B114; GenBank accession no. DQ105654) isolated from Guangzhou, China, in 2004. Because of the high sequence identity to the Gomen strain (HAdV-B7 prototype strain; GenBank accession no. AY594255), the fiber gene of the BJ-2024–563/2019 strain likely originated from a prototype-like strain of HAdV-B7, whereas the fiber gene of the BJ-2024–565/2019 strain was more closely related to the 0901HZ/ShX/CHN/2009 strain isolated in Shaanxi, China in 2009 (HAdV-B7; GenBank accession no. JF800905) (Figures 2, 3). Those results corroborated the phylogenetic findings and supported the designation of the 2 strains as a novel recombinant genotype, HAdV-B117 (P7H3F7).

### Variation Analysis in aa

Compared with the parental Guangzhou02 strain, both novel strains had 3 aa substitutions in the penton base protein: V128I, G163D, and A482T, but none in the RGD Loop region (Appendix Figure 2, panel A). Homology modeling revealed no major structural differences at these substitution sites between the Guangzhou02 strain and the novel strains. Within HAdV-B, the RGD Loop region was the most variable region of Penton base protein (Appendix Figure 2, panel A).

For hexon protein, both novel strains had 9 aa substitutions (R18Q, A99T, N155G, R156E, E197D, V239M, A289D, A454D, and H563Y), 8 aa deletions (T194, E195, G196, T265, G290, T446, D452, and D453), and 1 aa insertion (321D) relative to the Guangzhou02 strain. In addition, the BJ-2024-563/2019 strain had 4 unique substitutions (D157E, N158R, T193E, and E198N), whereas the BJ-2024-565/2019 strain had 3 (T193A, E198K, and V288A) (Appendix Figure 2, panel B). Homology models of both strains were built and superimposed with that of the Guangzhou02 strain to visualize the structural variations (Appendix Figure 2, panels C, D). The root mean square deviation for the 2 superimposed structures were both 0.029. Disparities were observed at the deletion sites in the Loop1 and Loop2 regions. However, the functional implications of those alterations warrant further study.

For the fiber protein, both novel strains exhibited identical 7 aa substitutions (T2A, V5A, D9T, T22N, S23L, Y353S, and R356T) compared with their parental strains, 2 of which (Y353S and R356T) were in the Knob region (Appendix Figure 2, panel E). Homology modeling indicated no major structural differences at those sites. Within HAdV-B, Fiber protein exhibited high variability across all regions (Appendix Figure 2, panel E).

## Discussion

This molecular epidemiologic investigation revealed that HAdV-B114 and HAdV-B7 were the most common genotypes of HAdVs in children hospitalized with acute LRTI in Beijing, China, during 2014–2024. However, previous studies have reported that HAdV-B3 and HAdV-B7 were the predominant genotypes among children with respiratory tract infections in northern China (*14*,*15*). Recently, a study found that a multitude of complete genome sequences from China labeled as HAdV-B3 from the past 2 decades should be considered as HAdV-B114 (P7H3F3) (*16*), suggesting major HAdV-B114 circulation in China over the past 2 decades. Those findings revealed that HAdV-B114 might have replaced HAdV-B3 as a dominant HAdV genotype for respiratory tract infections among children in northern China.

In this study, a novel HAdV genotype, HAdV-B117 (P7H3F7), was isolated from 2 children hospitalized with severe CAP. WGS, phylogenetic, and recombination analyses confirmed that HAdV-B117 was a novel intertypic recombinant of species HAdV-B. Its genomic backbone was derived from HAdV-B114 (P7H3F3), with the fiber gene acquired from HAdV-B7 (P7H7F7).

The 2 patients, a boy and a girl both <5 years of age, aligned with the typical age profile of HAdV susceptibility (*17*,*18*). One resided in Beijing and was admitted to the general ward on December 19, 2019; the other came from Hebei Province and entered the respiratory ward on December 26, 2019. Our findings indicate that HAdV-B117 was detected in 2 different areas in China, suggesting the potential for a new epidemic in the future. Both patients’ symptoms included high fever, cough, and sputum secretion, which were hallmarks of HAdV respiratory infections, and both patients were diagnosed with severe CAP accompanied with serious complications. The clinical manifestations resembled severe HAdV-B3 or HAdV-B7 pneumonia cases previously reported and indicate a potentially severe phenotype of HAdV-B117 infection. Increased vigilance and enhanced surveillance for HAdV-B117 are warranted. The inflammatory markers of 1 child who had a patent foramen ovale were extremely elevated, and bacterial co-infection could not be ruled out. Both children eventually recovered.

Recombination is a hallmark of HAdV evolution and a primary driver of new genotype emergence (*19*,*20*). HAdV-53–116 genotypes were intertypic recombinants (http://hadvwg.gmu.edu). HAdV-B117 also exemplified that process, integrating the fiber gene of HAdV-B7 into HAdV-B114 backbone. Because the fiber protein mediates viral attachment to host cells and the fiber gene of HAdV-B117 was from HAdV-B7, HAdV-B117 might share tropism and infectivity traits with HAdV-B7 and become a critical pathogen of acute respiratory infections.

Although HAdV-B117 was isolated from 2 patients with CAP in different regions of China, the prevalence and potential effect on public health of HAdV-B117 are not clear and need further monitoring. The hexon protein is the main antigenic protein of HAdV and induces the host immune response (*21*). The hexon gene of HAdV-B117 came from HAdV-B114 and the hexon gene of HAdV-B114 was from HAdV-B3. Comparative analysis revealed that the hexon protein of HAdV-B117 was highly similar to its parental strain (HAdV-B114), with nearly identical aa sequences. Therefore, HAdV-B114 and HAdV-B117 might exhibit similar antigenic characteristics as HAdV-B3, suggesting that exposure to HAdV-B3/114 might provide protective cross-immunity against HAdV-B117, which requires further experimental validation. However, previous studies have reported that receptor-binding regions in the knob region of the fiber protein could also elicit neutralizing antibodies (*22*,*23*). The fiber gene of HAdV-B117 originated from HAdV-B7 and was different from that of HAdV-B3/114, so the cross-immunity generated by HAdV-B3/114 might not be sufficient to block its transmission. Furthermore, previous studies demonstrated low levels of herd immunity against HAdV-B3 and HAdV-B7 in children from China (*24*,*25*), indicating that HAdV-B117 might cause potential epidemics in this population. Of note, mutations were also observed in the loop 1 and loop 2 regions of the hexon protein and the knob region of the fiber protein of HAdV-B117. Homology modeling revealed that aa deletions in the loop 1 and loop 2 regions of the hexon protein might alter protein structure, potentially impairing neutralizing antibody production and binding and enabling immune evasion, which merits further investigation. In addition, the replication dynamics of HAdV-B117 resembled those of currently prevalent HAdV-B genotypes, including HAdV-B3, HAdV-B7, HAdV-B14, and HAdV-B55 (data not shown), which were associated with severe infections and outbreaks (*26*,*27*). Those findings indicate that HAdV-B117 has the potential to cause epidemic outbreaks of respiratory disease and further monitoring is needed.

HAdV-B7 was generally considered to have higher replication, virulence, and infectivity than HAdV-B3 (*4*,*28*). Whole-genome comparative analysis revealed that the primary differences between HAdV-B3 and HAdV-B7 were in the fiber protein (*29*,*30*). A previous study (*31*) found that the higher binding affinities of fiber protein’s knob region to cellular receptors resulted in greater infectivity and virulence of HAdV-B7 and HAdV-B55 than those of HAdV-B3, thereby increasing their pathogenicity. HAdV-B117 acquired the fiber gene from HAdV-B7 through recombination; therefore, further monitoring of the virulence, infectivity, and pathogenicity of HAdV-B117 is needed.

In conclusion, this study identified a novel recombinant genotype within species HAdV-B, HAdV-B117 (P7H3F7), isolated from 2 pediatric cases of severe respiratory illness. In vitro, the replication kinetics of HAdV-B117 were similar to those of HAdV-B3 and HAdV-B7. Future research on its biological characteristics and epidemiology is needed. The emergence of this novel recombinant highlights the need for continuous genomic surveillance of HAdV.

Appendix Additional information about identification of novel recombinant human adenovirus genotype B117 from pediatric cases, China.
