# Supplementary material for: Identification of Novel Recombinant Human Adenovirus Genotype B117 from Pediatric Cases, China
Source: Emerg Infect Dis. 2026 Jun;32(6):950–8. doi: 10.3201/eid3206.250940 (PMC13245220; doi:10.3201/eid3206.250940)
Supplement: Appendix — Additional information about identification of novel recombinant human adenovirus genotype B117 from pediatric cases, China. [file 25-0940-Techapp-s1.pdf]

Article DOI: https://doi.org/10.3201/eid3206.250940

EID cannot ensure accessibility for supplementary materials supplied by authors. Readers who have difficulty accessing supplementary content should contact the authors for assistance.

Identification of Novel Recombinant Human Adenovirus Genotype B117 from Pediatric Cases, China

Appendix

Appendix Table 1. Clinical characteristics of 2 patients with HAdV*-B117 infection

**Sample name**	**BJ-2024-563/2019**	**BJ-2024-565/2019**
**Clinical characteristics**		
Age	4 years old	3 years old
Gender	Male	Female
Occupation	Kindergarten children	Kindergarten children
Permanent residence	Beijing	Hebei
Admission time	2019/12/19	2019/12/26
Ward	General ward	Respiratory ward
underlying diseases	Allergic rhinitis	Patent foramen ovale
Fever	40℃	40.7℃
Cough	Yes	Yes
Sputum	Yes	Yes
Wheezing	No	No
Dyspnea	No	No
Tachypnea	No	Yes
White blood cells ($\times 10^9$/L)	6.35	6.47
Neutrophils ($\times 10^9$/L)	2.05	1.99
Lymphocytes ($\times 10^9$/L)	3.3	2.92
Red blood cells ($\times 10^{12}$/L)	6.09	3.5
Hemoglobin (g/L)	109	91
Platelets ($\times 10^9$/L)	661	612
Procalcitonin (ng/ml)	/	5.561
C reactive protein (mg/L)	10	65
Alanine aminotransferase (U/L)	390.9	83.2

Sample name<br>Clinical characteristics	BJ-2024-563/2019	BJ-2024-565/2019
Aspartate aminotransferase (U/L)	467	62.3
Creatine kinase (U/L)	398	481
Creatine Kinase-MB (U/L)	15	27
Lactate dehydrogenase (U/L)	601	618
D-dimer (mg/L)	/	0.862
Ferritin (ng/ml)	/	600
ESR* (mm/h)	25	100
Electrocardiogram	Sinus arrhythmia, sinus bradycardia	Sinus arrhythmia, prolonged PR interval
Echocardiography	No abnormality	Patent foramen ovale
Chest X-ray	The markings of both lungs were thick and fuzzy, and patchy shadows could be seen in the inner zone of the both lower lungs	The markings of both lungs were thick and fuzzy, and patchy shadows could be seen in both lungs
Chest computed tomography	Patchy high-density shadows could be seen in both lungs, most prominently in the middle lobes of the right lung	Patchy and small nodular dense opacities could be seen in both lungs, with air bronchograms observed within some of the lesions
Diagnosis	Severe community-acquired pneumonia	Severe community-acquired pneumonia
History of antibiotic use	Azithromycin, Cefotaxime Sodium/Sulbactam	Ceftriaxone, Azithromycin
Immunotherapy	Methylprednisolone, Intravenous immunoglobulin	Methylprednisolone, Intravenous immunoglobulin
PICU* admission	No	No
Hospitalization	8 days	11 days
Complications	Liver function damage, Atelectasis, Sinus bradycardia	Liver function damage, Myocardial damage, Coagulopathy
Outcome	Improved and discharged	Improved and discharged

*HAdV, human adenovirus; ESR, erythrocyte sedimentation rate; PICU, pediatric intensive care unit.

Appendix Table 2. Reference sequences used in this study

Serial number	Accession number	Location of isolation	Year of isolation	Sequences	Genotype
1	AY599834	USA	1953	Complete genome and Penton base, Hexon, Fiber, E1-E4 gene	HAdV*-B3
2	AY594255	USA	1954	Complete genome and Penton base, Hexon, Fiber, E1-E4 gene	HAdV-B7
3	JF800905	Chinese mainland	2009	Complete genome and Penton base, Hexon, Fiber, E1-E4 gene	HAdV-B7
4	AY163756	USA	1954	Complete genome and Penton base, Hexon, Fiber, E1-E4 gene	HAdV-B11
5	MG905111	Netherlands	1957	Complete genome and Penton base, Hexon, Fiber, E1-E4 gene	HAdV-B14
6	AY601636	Saudi Arabia	1957	Complete genome and Penton base, Hexon, Fiber, E1-E4 gene	HAdV-B16
7	AY601633	Saudi Arabia	1957	Complete genome and Penton base, Hexon, Fiber, E1-E4 gene	HAdV-B21
8	AY737797	USA	1972	Complete genome and Penton base, Hexon, Fiber, E1-E4 gene	HAdV-B34
9	AY128640	USA	1973	Complete genome and Penton base, Hexon, Fiber, E1-E4 gene	HAdV-B35
10	AY737798	Netherlands	1988	Complete genome and Penton base, Hexon, Fiber, E1-E4 gene	HAdV-B50
11	FJ643676	Chinese mainland	2006	Complete genome and Penton base, Hexon, Fiber, E1-E4 gene	HAdV-B55
12	JN860676	Argentina	1987	Complete genome and Penton base, Hexon, Fiber, E1-E4 gene	HAdV-B66
13	JN860678	Argentina	2004	Complete genome and Penton base, Hexon, Fiber, E1-E4 gene	HAdV-B68
14	KF633445	Germany	Unknown	Complete genome and Penton base, Hexon, Fiber, E1-E4 gene	HAdV-B76
15	KF268328	Germany	1985	Complete genome and Penton base, Hexon, Fiber, E1-E4 gene	HAdV-B77
16	KT970441	USA	2013	Complete genome and Penton base, Hexon, Fiber, E1-E4 gene	HAdV-B78
17	LC177352	Japan	2015	Complete genome and Penton base, Hexon, Fiber, E1-E4 gene	HAdV-B79
18	OR735193	USA	2012	Complete genome and Penton base, Hexon, Fiber, E1-E4 gene	HAdV-B106
19	AY599836	USA	1997	Complete genome and Penton base, Hexon, Fiber, E1-E4 gene	HAdV-B114
20	DQ105654	Chinese mainland	2004	Complete genome and Penton base, Hexon, Fiber, E1-E4 gene	HAdV-B114

*HAdV, human adenovirus.

Appendix Table 3. The recombination events identified by RDP4*

Breakpoint Positions		Recombinant	Minor Parental	Major Parental	Detection Methods						
Begin	End	Sequence(s)	Sequence(s)	Sequence(s)	RDP	GENECONV	Bootscan	Maxchi	Chimaera	SiSscan	3Seq
94†	134	BJ-2024–563/2019	AY594255/HAdV-B7	DQ105654/HAdV-B114	NS	4.59E-12	1.43E-6	4.08E-3	4.00E-3	NS	3.73E-6
29,824†	30,853	BJ-2024–563/2019	AY737798/HAdV-B50	DQ105654/HAdV-B114	5.21E-40	2.37E-16	NS	4.28E-11	4.75E-11	8.39E-8	2.66E-15
31,395	32,379	BJ-2024–563/2019	AY594255/HAdV-B7	DQ105654/HAdV-B114	6.35E-128	2.41E-117	5.73E-123	6.64E-42	1.11E-41	1.21E-51	8.88E-16
29,831	30,859†	BJ-2024–565/2019	AY737798/HAdV-B50	DQ105654/HAdV-B114	4.03E-26	1.06E-11	NS	1.58E-11	1.72E-11	1.56E-8	8.88E-16
31,401	32,385	BJ-2024–565/2019	JF800905/HAdV-B7	DQ105654/HAdV-B114	7.53E-137	2.81E-129	6.83E-132	2.46E-43	9.79E-43	3.65E-52	8.88E-16

*Breakpoint positions are in recombinant sequence. Minor parent, parent contributing the smaller fraction of sequence; major parent, parent contributing the larger fraction of sequence. HAdV, human adenovirus; NS: not significant

†The actual breakpoint position is undetermined.

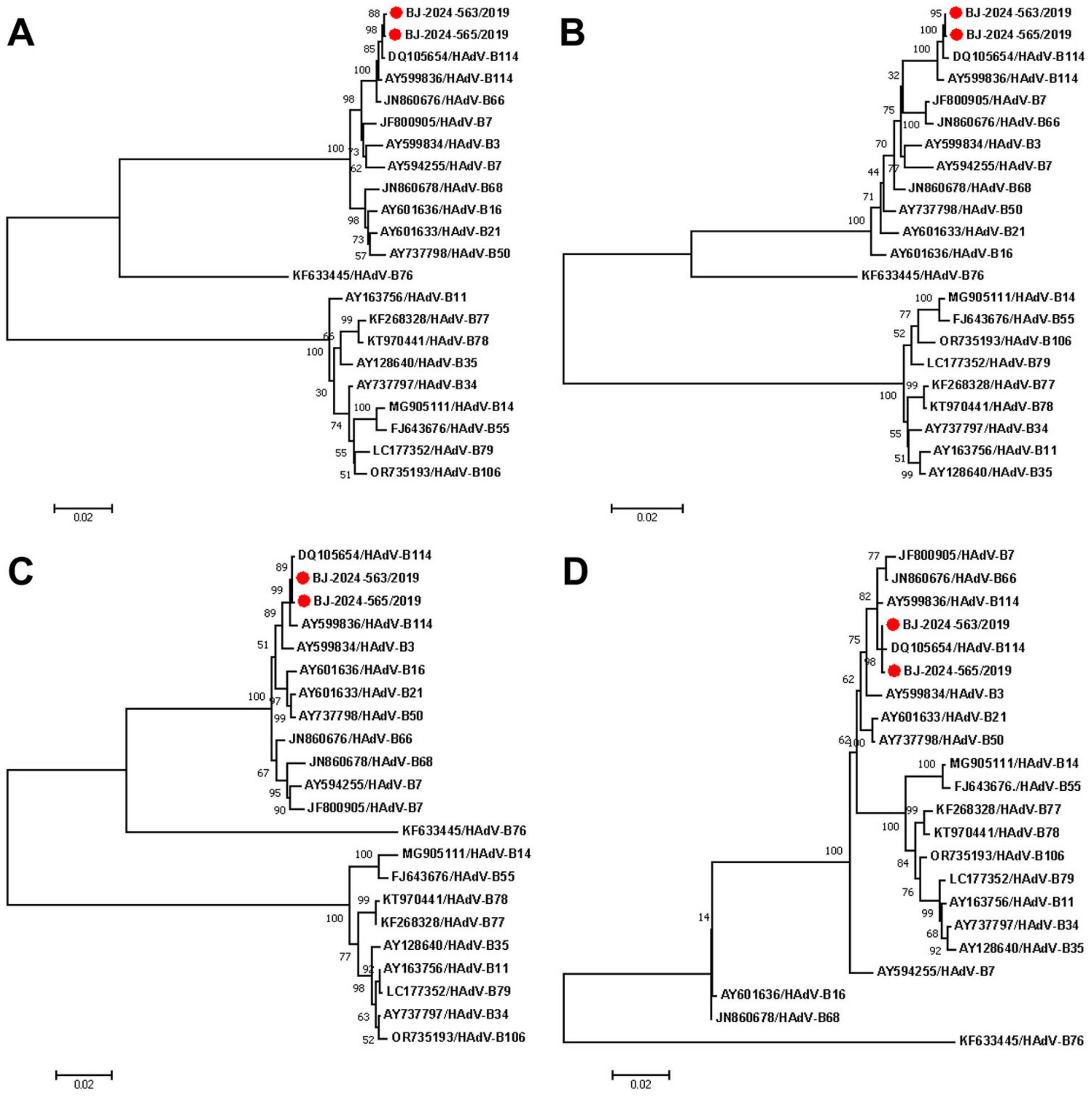

Appendix Figure 1. Phylogenetic analysis of the *E1*-*E4* genes of two novel strains and other species HAdV-B genotypes. The phylogenetic tree of (A) the *E1* gene was constructed by the Maximum Likelihood method and the Tamura-Nei model using 1,000 replicates. The phylogenetic trees of (B) *E2*, (C) *E3* and (D) *E4* genes were constructed by the Maximum Likelihood method and the Hasegawa-Kishino-Yano model using 1,000 replicates. Red dots indicate the two novel strains obtained in this study. HAdV, human adenovirus.

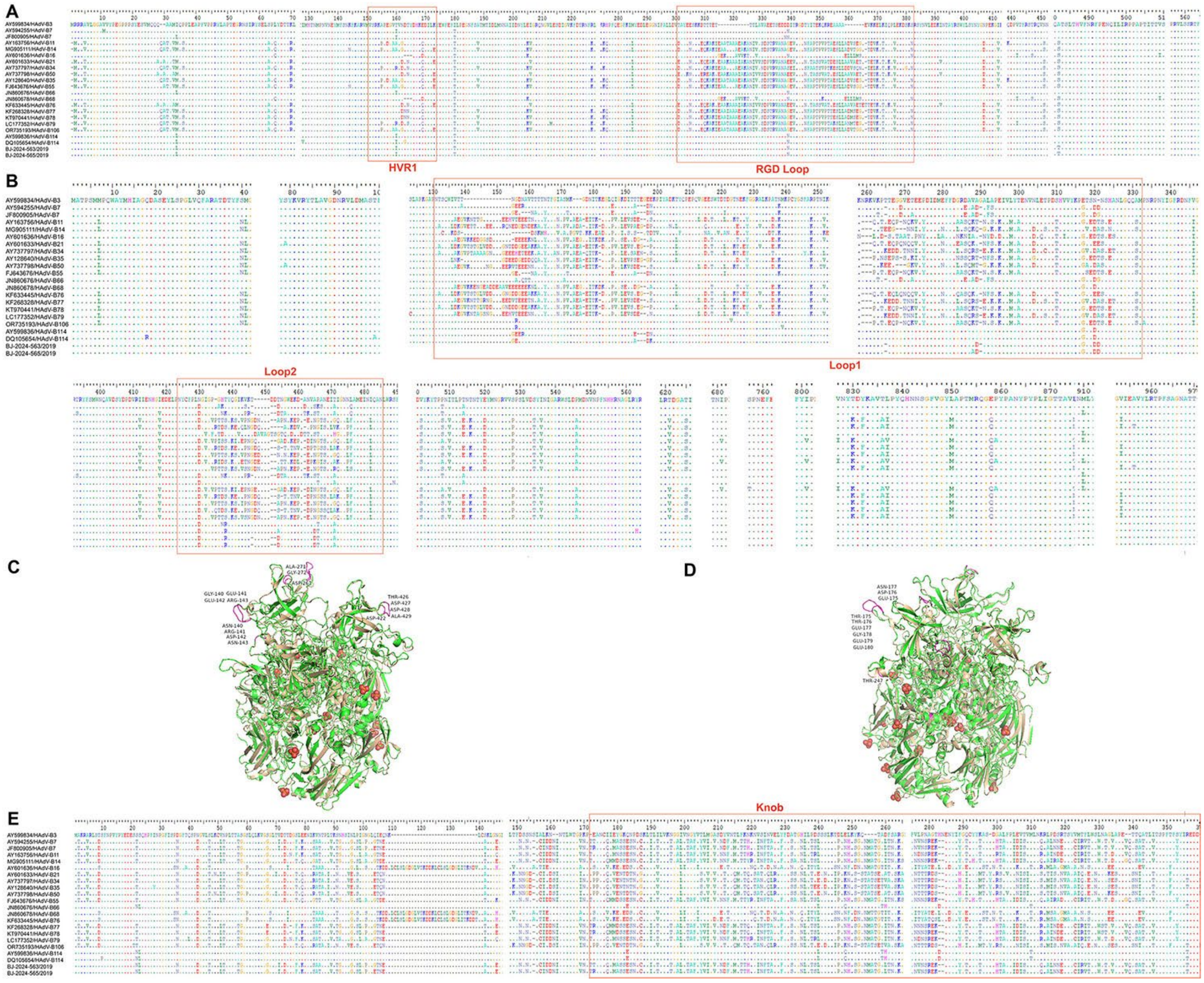

Appendix Figure 2. Amino acid variation analysis of 3 major capsid proteins of 2 novel strains and other species HAdV-B genotypes. Amino acid mutations of (A) Penton base, (B) Hexon and (E) Fiber proteins. C and D) Homology modeling of Hexon protein with the Guangzhou02 strain (green) superimposed over the BJ-2024–563/2019 strain (wheat). The amino acid mutation sites are indicated in pink. Because the amino acid deletion sites causing protein structure changes were in the same positions of Hexon protein of the two novel strains, the BJ-2024–563/2019 strain was selected for homology modeling. HAdV, human adenovirus.
